# The complete mitochondrial genome of sponge *Halichondria* (*Halichondria*) sp. (Demospongiae, Suberitida, Halichondriidae)

**DOI:** 10.1080/23802359.2016.1192516

**Published:** 2016-07-12

**Authors:** Dexiang Wang, Yuan Zhang, Dan Huang

**Affiliations:** State-Province Joint Engineering Laboratory of Marine Bioproducts and Technology, Xiamen University, Xiamen, China

**Keywords:** Demosponge, *Halichondria (Halichondria)* sp., mitogenome, sponge

## Abstract

In this study, we found out the complete mitochondrial genome of *Halichondria* (*Halichondria*) sp., a common demosponge in China. This is the first complete mitochondrial report on the genus *Halichondria*. The mitochondrial genome of *Halichondria* (*Halichondria*) sp. is 20 746 bp in length, with 14 protein-coding genes, two rRNA genes and 25 tRNA genes. When compared with the complete mitochondrial genome of *Hymeniacidon sinapium*, our phylogenetic result suggested that *Halichondria* (*Halichondria*) sp. converged well according to morphological result.

Sponge systematics is a long-standing issue due to vulnerability to environmental modification, which enhancing the difficulty in comparing taxonomy to the dozens of species in this genus or subgenus. As a supplementary measure for morphological taxonomy, studies have applied the mitochondrial genome of metazoa as a marker to resolve taxonomic controversies (Gissi et al. 2008). *Halichondria* (*Halichondria*) sp., a representative sponge in the coastal waters of Fujian province, China, belong to the family Halichondriidae (Gray 1867). Currently, no mitogenome was recorded for genus *Halichondria*. In this study, we focused on exploring a molecular alignment approach of the mitochondrial genome to supplement the mitogenome database of demosponges and figure out the molecular relationship among them.

Specimen was collected from Gulei Peninsula, Fujian Province, China (117.5928E 23.8015N), in May, 2014 and deposited in the Museum of Marine Science and Technology, Xiamen University with voucher number of XMU02_001 078. DNA was extracted with the method adapted from guanidinium isothiocyanate (Wilson & Carson 2001) and sequenced with MPS (massive parallel sequencing) Illumina technology. Mitogenome was constructed in a paired-end library with an insert size of 420 bp and sequenced using a Hiseq2500 by PE125 strategy. Sequencing and annotation was performed at Beijing Novogene Bioinformatics Technology Co. Ltd., (Beijing, China). Clean reads were assembled by SOAPdenovo (Li et al. 2010, Li et al. 2008) to produce a single circular form of the complete mitochondrial genome. A whole genome Blast (Altschul et al. 1990) search (E-value < = 1e-5, minimal alignment length percentage > = 40%) was conducted against 6 databases. An alignment of the assembled scaffold for *Halichondria (Halichondria)* sp. demonstrated the mitogenome of *Hymeniacidon sinapium* (GenBank number: KF192342.1) shared many similarities (90% identity) with our sample. We used the homological mitogenome as reference using the mitochondrial genome annotation (MITOS) server (Bernt et al. 2013) for annotation and blastx to improve results. Subsequently, we obtained annotated Coding DNA Sequences (CDS), transfer RNA genes (tRNA) and ribosomal RNA (rRNA) genes.

The complete genome of *Halichondria* (*Halichondria*) sp. is 20 746 bp in length with 14 protein-coding genes, 25 tRNA genes and two rRNA subunits. The base composition of the genome in *Halichondria* (*Halichondria*) sp. is A (34.79%), T (29.54%), C (21.49%) and G (14.18%), with a GC content of 35.13% (Table 1). The annotated mitogenome has been submitted to NCBI (GenBank accession number KX244759).

**Table 1.** Mitochondrial genome organization of *Halichondria* (*Halichondria*) sp.

Name	From	To	Direction	Length (bp)
*trnP* (tgg)	659	733	–	75
*trnS* (gct)	790	864	–	75
*atp9*	899	1132	–	234
*trnT* (tgt)	1169	1241	–	73
*cob*	1315	2427	–	1113
*trnN* (gtt)	2496	2567	–	72
*trnW* (tca)	2662	2732	–	71
*trnL* (tag)	2746	2819	–	74
*trnQ* (ttg)	2859	2930	–	72
*cox3*	3148	3930	–	783
*trnR* (tct)	4017	4089	–	73
*atp6*	4119	4766	–	648
*atp8*	5012	5149	–	138
*trnK* (ttt)	5151	5223	–	73
*cox2*	5363	6025	–	663
*trnM* (cat)	6153	6224	–	72
*trnY* (gta)	6244	6315	–	72
*rrnL*	6477	9310	–	2834
*trnV* (tac)	9392	9464	–	73
*trnG* (tcc)	9489	9560	–	72
*rrnS*	9562	10925	–	1364
*trnF* (gaa)	10927	10999	–	73
*trnM* (cat)	11055	11126	–	72
*trnA* (tgc)	11189	11261	–	73
*nad5*	11308	13164	–	1857
*nad2*	13413	14408	–	996
*trnM* (cat)	14858	14928	–	71
*trnI* (gat)	15011	15084	–	74
*trnL* (taa)	15088	15167	–	80
*nad1*	15201	16187	–	987
*trnC* (gca)	16233	16306	–	74
*trnS* (tga)	16379	16454	–	76
*cox1*	16538	18079	–	1542
*nad4l*	18219	18476	–	258
*trnR* (tcg)	18510	18581	–	72
*nad3*	18620	18949	–	330
*trnD* (gtc)	19013	19084	–	72
*nad6*	19133	19684	–	552
*trnE* (ttc)	19685	19757	–	73
*trnH* (gtg)	19808	19879	–	72
*nad4*	20003	20728	–	726

Direction: “+” stands for 5'to 3', “–” stands for 3'to 5'.

The complete mitochondrial genome of *Hymeniacidon sinapium* most closely matches that of *Halichondria (Halichondria)* sp. Both of them belong to the same family Halichondriidae, yet according to morphological taxonomy, they are not the same genus. After multiple sequence alignment with other mitogenomes of demosponges by MAFFT (Katoh & Standley 2013), we constructed a phylogenetic tree (Figure 1) using Maximum Likelihood (ML) method in MEGA 6.06 (Tamura et al. 2013), which converged well according to morphological result. To conclude, the mitogenome study of *Halichondria* (*Halichondria*) sp. may contribute to a better phylogenetic understanding of demosponges, especially using as a supplemental means to attribute the controversial species in the near future.

**Figure 1.**
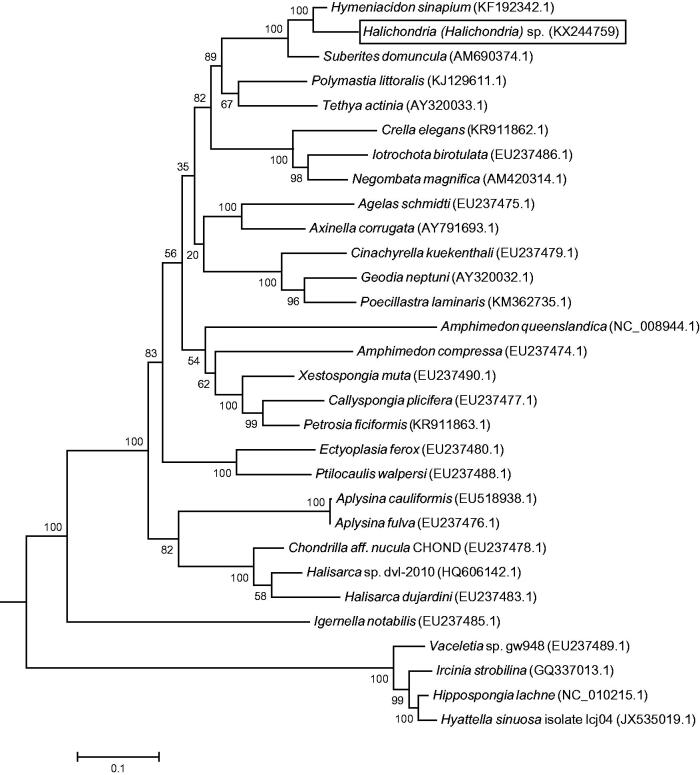
The consensus phylogenetic relationship of *Halichondria (Halichondria)* sp. and the other species of Demospongiae from Maximum Likelihood (ML) analysis with 1000 bootstrap. The number on the branches are the bootstrap values for ML. The Genbank accession numbers of each species are shown in the brackets.
